# Alterations in Morphology and Adult Neurogenesis in the Dentate Gyrus of *Patched1* Heterozygous Mice

**DOI:** 10.3389/fnmol.2018.00168

**Published:** 2018-05-23

**Authors:** Francesca Antonelli, Arianna Casciati, Mirella Tanori, Barbara Tanno, Maria V. Linares-Vidal, Noemi Serra, Monserrat Bellés, Alessandro Pannicelli, Anna Saran, Simonetta Pazzaglia

**Affiliations:** ^1^Laboratory of Biomedical Technologies, Agenzia Nazionale per le Nuove Tecnologie, l’Energia e lo Sviluppo Economico Sostenibile (ENEA), Rome, Italy; ^2^Laboratory of Toxicology and Environmental Health, School of Medicine, Institut d’Investigació Sanitària Pere Virgili (IISPV), Rovira I Virgili University (URV), Reus, Spain; ^3^Physiology Unit, School of Medicine, Institut d’Investigació Sanitària Pere Virgili (IISPV), Universitat Rovira i Virgili, Tarragona, Spain; ^4^Technical Unit of Energetic Efficiency, Agenzia Nazionale per le Nuove Tecnologie, l’Energia e lo Sviluppo Economico Sostenibile (ENEA), Rome, Italy

**Keywords:** sonic hedgehog pathway, hippocampal neurogenesis and neuronal lineage differentiation, notch pathway, TLX nuclear receptor, expression profiles of neurogenesis-related genes, behavioral effects

## Abstract

Many genes controlling neuronal development also regulate adult neurogenesis. We investigated *in vivo* the effect of Sonic hedgehog (Shh) signaling activation on patterning and neurogenesis of the hippocampus and behavior of *Patched1* (*Ptch1*) heterozygous mice (*Ptch1^+/−^*). We demonstrated for the first time, that *Ptch1^+/−^* mice exhibit morphological, cellular and molecular alterations in the dentate gyrus (DG), including elongation and reduced width of the DG as well as deregulations at multiple steps during lineage progression from neural stem cells to neurons. By using stage-specific cellular markers, we detected reduction of quiescent stem cells, newborn neurons and astrocytes and accumulation of proliferating intermediate progenitors, indicative of defects in the dynamic transition among neural stages. Phenotypic alterations in *Ptch1^+/−^* mice were accompanied by expression changes in Notch pathway downstream components and *TLX* nuclear receptor, as well as perturbations in inflammatory and synaptic networks and mouse behavior, pointing to complex biological interactions and highlighting cooperation between Shh and Notch signaling in the regulation of neurogenesis.

## Introduction

The Hedgehog signaling pathway is required for multiple aspects of embryonic development of a wide range of tissues, both in invertebrates and vertebrates (McMahon et al., 2003). Sonic hedgehog (Shh) is a secreted glycoprotein that signals through its receptors Patched1 (Ptch1) and Smoothened (Smo), leading to activation of zinc-finger Gli transcription factors (Ruiz i Altaba et al., 2002).

In the mammalian central nervous system (CNS), Shh is expressed in the ventral neural tube at early embryonic stages and is essential to establish the ventral pattern along the neuroaxis. At later embryonic stages other brain regions such as cerebellum, amygdala, dentate gyrus (DG) of the hippocampus, olfactory bulb and neocortex, also begin to express Shh (Ruiz i Altaba et al., 2002).

Shh is also a key factor for the formation of the neural germinal niches located in the subventricular zone (SVZ) of the lateral ventricles and the subgranular zone (SGZ) of the hippocampal DG (Ihrie and Alvarez-Buylla, 2008; Zhao et al., 2008). Direct proofs of its involvement in establishing the neurogenic niches comes from mouse studies in which *Shh* or *Smo* were ablated causing several brain abnormalities including reduced number of progenitors in the SVZ and SGZ (Machold et al., 2003). Also, genetic ablation of primary cilium, essential for Shh signaling, results in decreased Shh target gene expression and a phenotype similar to that of Smo-deficient mice (Breunig et al., 2008; Han et al., 2008). Instead, expression of a constitutively active *Smo* (*SmoM2*-*YFP*) resulted in a marked expansion of the DG (Han et al., 2008).

The neurogenic niche, comprising neural stem/precursor cells, immature and mature neurons, other glial cells, endothelia, astrocytes, microglia and extracellular matrix, constitutes an integrated multicellular neural system. This system provides a great variety of signals, including Shh, that orchestrate the control of adult neurogenesis (Ahn and Joyner, 2005; Faigle and Song, 2013). Adult neural stem cells (NSCs), residing in the SGZ, are maintained in a largely quiescent state. Upon activation by niche-derived and intrinsic signals they undergo proliferation to generate proliferating transit amplifying progenitors (TAPs) that enlarge the pool of neurogenic cells, giving rise to immature neurons. These cells progress through neuronal differentiation to granule cell neurons that integrate into functional neuronal circuits. In the rodent brain, thousands of new neurons are generated daily, contributing to homeostasis and brain functions that underlie certain forms of learning and memories (Deng et al., 2010). Failing or altered neurogenesis has been associated with a number of neuropsychiatric diseases including anxiety and depression (Winner et al., 2011).

While the role of Shh pathway in development and tumorigenesis in CNS has been extensively established, we just begin to appreciate that Shh signaling continue to act in the adult brain, even though its functional role once the morphogenetic and proliferative processes are concluded is still unclear. There is growing interest in the identity of cells producing Shh ligand. High levels of Shh transcripts have been identified in basal forebrain, brainstem and cranial nerve nuclei (Traiffort et al., 1998, 1999; Machold et al., 2003). Instead, the germinal zones of the adult brain do not produce Shh ligand autonomously but contain Shh-responsive cells (Ahn and Joyner, 2005; Álvarez-Buylla and Ihrie, 2014) and Ptch1 and Smo have been detected in the DG (Traiffort et al., 1998, 1999). Furthermore, in the adult hippocampus Shh signaling pathway has been shown to regulate proliferation of NSCs (Lai et al., 2003) but its role as regulator of neurogenesis is still largely uncovered.

To study the *in vivo* effect of the Shh pathway activation on both hippocampal morphogenesis and neurogenesis, we used mice with germline inactivation of one copy of the *Ptch1* gene (Hahn et al., 1998). *Ptch1* homozygous germ-line inactivation is embryonic lethal, causing death by embryonic day 9.5 because of the defects in the developing nervous and cardiovascular system. Even *Ptch1* conditional inactivation at later embryonic stage (E14.5–E16.5) leads to rapid tumor formation with 100% medulloblastoma incidence by 3–4 weeks of age in glial fibrillary acid protein (GFAP)-Cre/Ptc^C/C^ mice (Yang et al., 2008). Instead, *Ptch1^+/−^* mice are viable, although predisposed to low incidence of spontaneous tumor in several tissues/organs, including the brain (Pazzaglia, 2006). Using this model, we investigated the effect of Shh constitutive activation on hippocampal neurogenesis, on behavior and the transcriptional consequences in genes regulating neurogenic program.

Our results show that constitutive activation of Shh pathway in *Ptch1^+/−^* mice causes morphological alterations of DG and defects in lineage progression with a deficit in newborn neurons. Expression changes in Notch downstream targets and in *TLX* nuclear receptor are also observed, suggestive of a crosstalk between Shh and Notch pathways in regulating the progression of neural stem/progenitor cells to neurons. Shh pathway deregulation also induces alterations in the inflammatory networks and synaptic functions that are reflected in behavioral changes. Mechanistic understanding of the cellular/molecular neurogenic process has clinical relevance, as altered neurogenesis is associated with a number of neuropsychiatric diseases.

## Materials and Methods

### Animals

Mice lacking one *Ptch1* allele (*Ptch1*^neo6-7/+^, named *Ptch1^+/−^* throughout the text) generated through disruption of exons 6 and 7 in 129/Sv embryonic stem cells and maintained on CD1 background were bred and genotyped as described (Hahn et al., 1998). Animals, all males to avoid gender variations, were housed under conventional conditions with food and water available *ad libitum* and a 12-h light cycle. All the experiments have been carried out in accordance to the Directive 2010/63/EU for animal experiments. Experimental protocols were reviewed by the Institutional Animal Care and Use Committee, and permission was issued by “Ministero della Salute” (Approval number is 365/2015-PR).

### Morphometric Analysis

**Figure 1:**
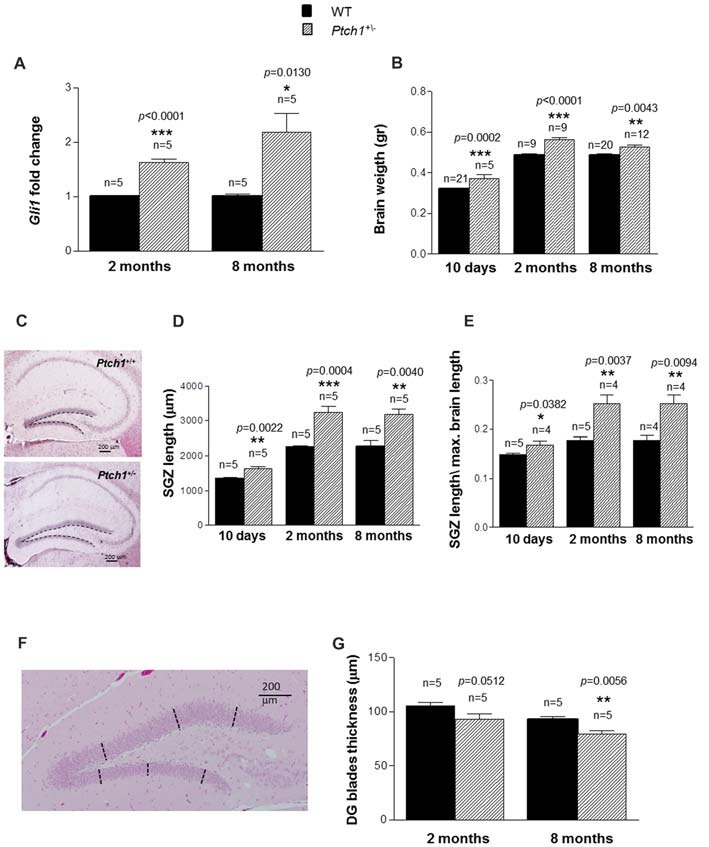
Sonic hedgehog (Shh) pathway deregulation leads to brain weight gain and dentate gyrus (DG) elongation. **(A)** Relative mRNA expression levels of *Gli1* in wild type (WT) and *Ptch1^+/−^* mice of 2 and 8 months of age. **(B)** Brain weight as a function of age and genotype. **(C)** Representative images of DG in WT and *Ptch1*^+/−^ mice (8 months of age), 4× objective, scale bar 200 μm. A line has been manually traced for each section to measure the subgranular zone (SGZ) length. **(D)** DG SGZ length as a function of age and genotype. **(E)** Ratio between mean DG SGZ length and maximum cross-sectional brain length in WT and *Ptch1*^+/−^ mice. **(F)** Representative image of DG (8 months of age), 4× objective, scale bar 200 μm. For determination of DG width, three perpendicular lines have been manually traced for each blade. **(G)** Thickness of DG blades as a function of age and genotype. The number of mice used per test is indicated in the graphs (n). Data are reported as mean ± standard error mean (SEM). *p < 0.05; **p < 0.01; ***p < 0.001 for comparison with controls (Student-*t* test).

For general morphometric analyses, wild type (WT) and *Ptch1^+/−^* mice, were sacrificed via cervical dislocations at 10 days, 2 and 8 months of age, and brains were excised and weighed before being fixed in 10% buffered formalin and embedded in paraffin wax for histological analysis (Tanori et al., 2013). To determine the SGZ length, serial sagittal sections were sampled every 100 μm through the whole cerebral hemispheres. The length of SGZ was measured for each section by manually tracing a line along the SGZ, as shown in Figure 1C. SGZ lengths were measured in each section and the mean value was expressed as the arithmetic mean measured out of 4–5 mice. For normalization, SGZ length was divided for the maximum length of cross-sectional brain. The thickness of DG blades was evaluated by measuring three randomly selected non-overlapping regions in medial, middle and lateral regions in the supra- and infrapyramidal blades after manually tracing six perpendicular lines as shown in Figure 1F. For morphometric analyses, the imaging software NIS-Elements BR 4.00.05 (Nikon Instruments Europe B.V., Italy) was used.

### Immunohistochemistry

Fixed tissue sections were immunostained as described (Casciati et al., 2016) using the following primary antibody diluted as indicated by manufacturer: GFAP (Z0334—Dako, Germany, 1:500), Sox2 (ab97959—Abcam, Germany, 1:500), doublecortin (DCX; 18723—Abcam, Germany, 1:2000), NeuN (MAB377—Millipore, Germany) and Iba1 (019-19741, Wako Pure Chemical Industries, Japan, 1:500). Cell quantification was performed on collected sections (stained for GFAP, Sox2, DCX) using the imaging software NIS-Elements BR 4.00.05 (Nikon Instruments Europe B.V., Italy). The number of positive cells in the SGZ was expressed per mm of the SGZ length. NSCs were counted based on criteria including SGZ localization, positive labeling and morphology. Immunohistochemical staining for NeuN was carried out in a rectangular field of 2000 μm^2^ in the supra- and infrapyramidal blade and in the crest area of the DG.

Quantitative analysis of astroglial cells (labeled by GFAP and Sox2 antibody) were identified in the molecular layer (ML) of the hippocampus and expressed as astrocytes/mm^2^. Statistical analyses were performed using GraphPad Prism 5.0. Statistical significance was determined using a two-tailed student’s *t*-test for comparison between pairs of means. *p*-values < 0.05 were considered to be statistically significant.

### Immunofluorescence Analysis

Brains were fixed for 22 h at 4°C in 4% paraformaldehyde and then washed twice in PBS, placed for 24 h in 30% sucrose solution, and frozen at 80°C in OCT. Sections of 15 μm were serially sliced on a cryostat. A standard immunostaining procedure was used for anti-Ki67 antibody detection. Briefly, cryosections were permeabilized in PBS containing 0.2% Triton X-100, blocked in 5% BSA and then incubated with the anti-Ki67 primary antibody (ab15580—Abcam, Germany, 1:1000) overnight at 4°C, washed and incubated with fluorescently tagged secondary antibody (Alexa Fluor 488, Invitrogen Corporation, Camarillo, CA, USA, 1:200). Cell quantification was performed as for immunohistochemical analysis.

### Real-Time and RT2 Profiler PCR Array

Brains (*n* = 5) were collected at 2 or 8 months of age and stored in RNA later over-night at 4°C. Then, DGs were manually dissected under a stereomicroscope according to the procedure illustrated in the video at https://www.jove.com/video/1543/dissection-of-hippocampal-dentate-gyrus-from-adult-mouse (Hagihara et al., 2009) and stored at −80°C in RNA later until RNA extraction. Two microgram of total RNA isolated using RNeasy Mini Kit (QIAGEN, Milan, Italy) were reverse transcribed with High-Capacity cDNA Reverse Transcription Kit (Applied Biosystems, Foster City, CA, USA). qRT PCR for *Gli1, TLX* and *Cyclin D1* was carried out on StepOnePlus^TM^ Real-Time PCR System (Applied Biosystems), using Power SYBR^®^ Green PCR Master Mix (Applied Biosystems) with primers listed in Supplementary Table S1, according to manufacturer’s instructions. Relative gene expression was quantified using Glyceraldehyde-3-phosphate dehydrogenase (GAPDH) as house-keeping gene. The comparative Ct (ΔΔCt) method was used to calculate the relative expression level of target genes. Data represent the average of three independent experiments. To quantify gene expression of 84 genes of RT^2^ Profiler Mouse Neurogenesis PCR Array (PAMM-404Z, SABiosciences, Qiagen, Germany), total RNA (0.5 μg) from DGs of 8-month old mice (*n* = 5) was used on a StepOnePlus (Applied Biosystems) according to manufacturer’s instructions. Only Ct values <35 were included in the calculations. The relative expression of each mRNA was normalized using the equation 2^−ΔΔCt^. For each genotype, three replicates were carried out and a cut-off of ±1.3 was used to analyze data. Gene expression was related to the mean expression of all five housekeeping genes included in the array.

### Cognitive Tests

Behavioral analysis was carried out in *Ptch1^+/−^* mice and WT littermates at 4 months of age.

#### Open-Field (OF) Test

The open-field (OF) test is used to assess the anxiety-like and the activity levels of the animals. The OF consisted in a wooden square (47 cm × 47 cm) surrounded by a dark wall (40 cm). The area of the maze within 15 cm from the wall was considered as peripheral (Bellés et al., 2010; Vicens et al., 2011), while the rest as the central area. At the beginning of the test mice were placed in the center of the arena and allowed to move freely around the maze to explore the environment for 15 min. To remove olfactory cues from the previous animal, the apparatus was cleaned with 70% ethanol after each observation. The video tracking software Ethovision XT© (Noldus Information Technologies, Wageningen, Netherlands), was used to measure the distance traveled over the maze. Additionally, the number of rearings (vertical standing of mice on hind legs) were registered. During the behavioral testing, indirect lighting was used and lighting levels maintained at ≈100 lux (Lalonde and Strazielle, 2012; Heredia et al., 2015).

#### Elevated Plus Maze (EPM) Test

The elevated plus maze (EPM) test is used to assess anxiety-like levels in mice. The apparatus used has two closed arms (25 × 5 × 16 cm) across from each other and perpendicular to two open arms (25 × 5 × 0.5 cm), with a center platform (5 × 5 × 0.5 cm). In the open arms, the small wall (0.5 cm) is used to decrease the number of falls. The entire apparatus is 50 cm above the floor. Mice were transported to the testing room 30 min before the tests. At the start of the session each animal was placed in the central square allowed to freely explore the environment for 5 min. To remove olfactory cues from the previous animal, the apparatus was cleaned with 70% ethanol after each observation. Performance was recorded by a video camera and data analyzed with Ethovision XT© (Noldus Information Technologies) video tracking software to measure the time spent in open arms (Walf and Frye, 2007; Heredia et al., 2016). In addition, the number of head dips (downward movements of the head towards the floor) was registered.

#### Water Maze (WM) Test

To evaluate spatial learning and memory, animals were subjected to the water maze (WM) test (Morris, 1984). The WM consisted of a circular tank (diameter 1 m; height 60 cm), divided into four quadrants. An escape platform (diameter 10 cm) was located 1 cm below the surface of the water in the target quadrant. Animals performed five trials/day for three consecutive days. During each trial, mice were allowed 60 s to find the hidden platform and to remain on it for 30 s. If the animal failed to find the platform within 60 s, it was placed on it. For each mouse, the order of the starting positions was randomized throughout the day. Extra-maze clues were located around the pool to provide a spatial configuration of the task. An internal mobile wall was added to the maze to avoid proximal cues and prevent egocentrical learning, being the wall randomly moved between trials. This seems to increase Morris WM sensitivity (Ribes et al., 2008). At the end of the third acquisition day, retention of the task was assessed by a probe trial, which consisted of a 60 s free swim without the escape platform. An additional probe trial to evaluate the spatial memory of animals (retention) were performed 48 h after the last training day. Animal performance was recorded using a video camera placed above the maze. Data were analyzed by the video tracking program Ethovision XT© (Noldus Information Technologies). During the probe trial, total time spent in the target quadrant, as well as the time spent in other quadrants were also measured in order to compare the time spent searching in the target quadrant between groups.

#### Radial Arm Maze (RAM) Test

Radial arm maze (RAM) is used to evaluate spatial-working memory in rodents. The maze consists of a central square (20 cm diameter) and eight radial attached arms (6 cm × 35 cm). In the current study, animals were trained for 3 days (one trial/day) to collect a small food pellet placed at the end of each arm. To remove olfactory cues from the previous animal, the apparatus was cleaned with 70% ethanol after each observation. In order to increase their motivation for the task, mice had free access to water, but were deprived of food 12 h before the initial trial. Each trial started with the mouse in the central platform facing the same arm and ended when the animal had eaten all food rewards (8 food pellets), or after 10 min. The optimum strategy implies a minimum number of visits to empty arms. The time spent in the arms in each session was measured, as well as the number of incorrect arm choices (visits to the same arm more than once during a single test session), and the number of incorrect arm entries (animal visited an arm and did not eat the reward).

#### Cognitive Statistical Analysis

Data are reported as the mean ± standard error mean (SEM). Homogeneity of variances was analyzed using the Levene’s test. If variances were homogeneous, ANOVA was used followed by the Tukey *post hoc* test to evaluate all dose groups simultaneously. If the variances were not homogeneous, the Kruskal–Wallis test was used. Differences between groups were analyzed using the Mann–Whitney U-test. Moreover, the paired *t*-test was used to compare the two different time-point tested. The ANOVA test and *post hoc* analyses adjusted by Bonferroni’s correction were used to analyze the progression for repeated measures of parameters recorded by the Ethovision XT© software. The level of statistical significance for all tests was established at *p* < 0.05. All data were analyzed by means of the statistical package GraphPad Prism software (San Diego, CA, USA) version 5.01.

## Results

### Activation of Shh Pathway *in Vivo* Increases Brain Weight and DG Length

To confirm sustained Shh signaling activation in the DG of *Ptch1^+/−^* mice, we evaluated the expression levels of Gli1, a well established biomarker of the Shh pathway activity, in microdissected DG tissue. Compared to WT mice, the *Ptch1^+/−^* mice showed a significantly increased expression of Gli1 mRNA at both 2 (1.6-fold, *p* < 0.0001) and 8 months (2.2-fold, *p* = 0.0130) of age (Figure 1A), compatible with the activation of the Shh pathway. Accordingly, the brain of *Ptch1^+/−^* mice showed a significant increase in weight compared to WT mice, that was already detectable at 10 days of age (16%, *p* = 0.0002) and persisted at 2 (15%, *p* < 0.0001) and 8 months (8%, *p* = 0.0043) of age (Figure 1B). We next examined the hippocampus for morphological differences and we found that *Ptch1^+/−^* mice exhibit significantly longer blades of the DG compared to WT mice (21%, *p* = 0.0022; 45%, *p* = 0.0004 and 40%, *p* = 0.0040 at 10 days, 2 and 8 months of age respectively; Figures 1C,D). To exclude that elongation of the DG might merely be a consequence of the enlarged brain, the length of the DG was normalized for the maximum brain length and significant increases in DG length were confirmed for all the time-points in *Ptch1^+/−^* mice (*p* = 0.037 at 10 days, *p* = 0.005 at 2 months and *p* = 0.032 at 8 months of age; Figure 1E). Finally, we examined the thickness of the DG, by taking three measures (tracing perpendicular lines) in the upper and 3 in the lower blades of the DG (Figure 1F). We found a reduction of DG thickness in *Ptch1^+/−^* compared to WT mice that was almost statistically significant at 2 months (*p* = 0.0512) and very significant (*p* = 0.0056) at 8 months (Figure 1G). Taken together these results indicate that aberrant activation of the Shh pathway in *Ptch1^+/−^* mice results in abnormal brain development, such as brain weight increase and DG morphological alterations.

### Activation of Shh Pathway Alters the Cell Stage Composition of DG in Adult Mice

**Figure 2:**
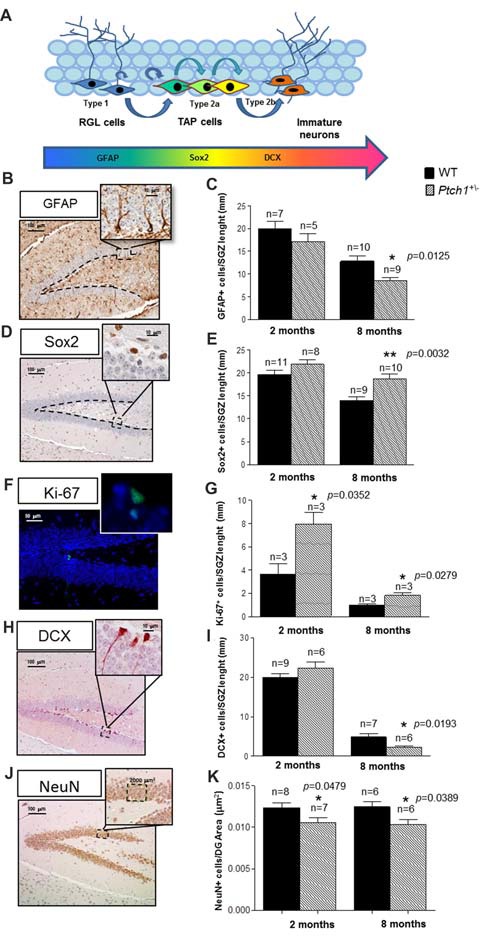
Shh pathway deregulation alters the cell stage composition of DG. **(A)** Schematic representation of adult neurogenesis in the hippocampal DG and relative stage specific markers. **(B,C)** Representative immunostaining images and quantification for glial fibrillary acidic protein (GFAP). **(D,E)** Sex determining region Y (SRY) box 2 (Sox2), **(F,G)** Ki67 and **(H,I)** doublecortin (DCX), **(J,K)** NeuN. Images, 10× magnification, scale bar = 100 μm; 20× magnification, scale bar = 50 μm; 100× magnification, scale bar = 10 μm. The number of mice used per test is indicated in the graphs (n). Data are reported as mean ± SEM *p < 0.05; **p < 0.01 for comparison with controls (Student-*t* test).

Adult neurogenesis in the DG requires the presence of long-lived NSCs, descending from NSC that originate at late gestation from the ventral hippocampus under the control of Shh, and then relocate in the SGZ (Li et al., 2013). Although, Shh has been identified as a regulator of adult hippocampal neural stem cells (Lai et al., 2003), direct investigations on its role in adult neurogenesis are still lacking. Adult hippocampal neurogenesis is a multistep process that generates only one type of neuron, i.e., granule cells in the DG. The prevailing model of adult hippocampal neurogenesis, summarized in Figure 2A, suggests that the largely quiescent NSCs, also called radial glial-like cells (RGLs, type-1 cells), pass through several developmental stages to become a granule neuron and fully integrate in the hippocampus neuronal network (Kempermann et al., 2015).

To investigate the role of Shh pathway in adult hippocampal neurogenesis *in vivo* and to detect possible Shh-dependent modification in the cellular composition of the SGZ of the DG, we applied criteria based on morphological cellular features and labeling of stage-specific adult neurogenesis markers following immunohistochemical analysis. Slowly dividing/quiescent RGLs, featured with a single radial process that extends through the granular cell layer of the DG, express GFAP. RGLs give rise to TAPs that are small round cells, endowed with high proliferative activity (Ki-67), expressing sex determining region Y (SRY) box 2 (Sox2^+^), that enlarge the pool of neurogenic cells (type 2a). The first indications of neuronal lineage choice appear at the level of type 2a cells and involves the suppression of *Sox2* expression, critical for the transition from stem/progenitor cells to newborn neurons labeled by DCX^+^ (type 2b cells). Subsequently, newborn neurons progress into mature granule neurons that finally integrate in the existing neuronal circuit. As expected, compared to 2 months-old mice, mice at 8 months showed a decrease of all the cell compartments labeled by GFAP, Sox2, Ki-67 or DCX (Figures 2B–I), indicative of an age-dependent physiological decline of neurogenesis, unrelated to Shh activation. Comparison of stage-specific cellular-markers in the DG of *Ptch1*^+/−^ and age-matching WT mice indicates that Shh-dependent perturbations of adult hippocampal neurogenesis are already evident at 2 months, as an increase in proliferation (*p* = 0.0352, Figure 2G) and a decrease in the mature neurons density (*p* = 0.0479, Figures 2J,K). Neurogenesis alterations in *Ptch*^+/−^ mice accumulate progressively with age, causing major changes at 8 months, that include depletion of type-1 RGL cells (GFAP^+^; *p* = 0.0125, Figure 2C) and accumulation of type 2a cells (Sox2^+^; *p* = 0.0032, Figure 2E), suggestive of an enlarged TAPs pool. They also showed a concordant increase in proliferating cells (Ki-67^+^; *p* = 0.0279, Figure 2G), as well as a decrease in the number of newly generated type 2b neurons (DCX^+^; *p* = 0.0193, Figure 2I) and in the mature neurons density (NeuN; *p* = 0.0389, Figure 2K), denoting concomitant alterations in the balance between self-renewal and differentiation.

Our findings demonstrate that constitutive activation of Shh pathway causes progressive defects in the dynamic transition among neural stages in the DG, including self-renewal, proliferation and differentiation. Taken together, our results point to a complex and multi-faced role for Shh in the hippocampus going from DG shaping at embryonic/neonatal age to the control of progression of NSCs into neurons during adulthood.

### Activation of Shh Pathway Alters Hippocampal Astroglia

**Figure 3:**
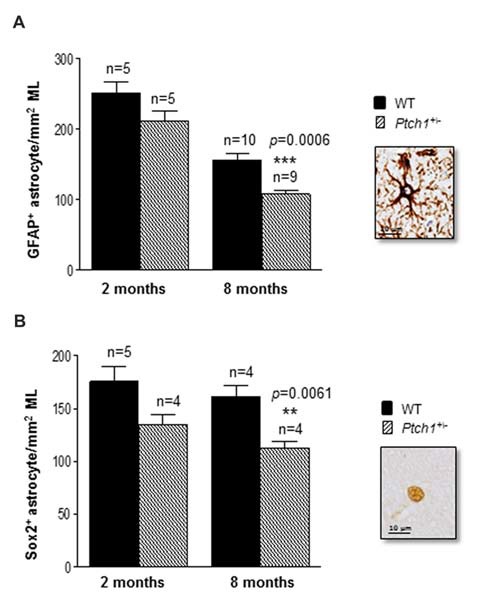
Shh pathway deregulation alters hippocampal astroglia. **(A)** Immunostaining and quantification of GFAP positive and **(B)** Sox2 positive astrocytes in the molecular layer (ML) of the hippocampus. Images, 100× magnification, scale bar = 10 μm. The number of mice used per test is indicated in the graphs (n). Data are reported as mean ± SEM **p < 0.01; ***p < 0.001 for comparison with controls (Student-*t* test).

Astrocytes provide trophic support for neurons, neuronal migration and inflammatory processes for maintenance of the neural network. Shh signaling has been suggested to regulate mature astrocytes in both the normal and injured brain (Garcia et al., 2010; Petrova et al., 2013; Sirko et al., 2013). To test whether the activation of the Shh pathway in *Ptch1^+/−^* mice also modulates the astrocyte production we evaluated the number of cells labeled by GFAP or Sox2 in the ML of the hippocampus (Figures 3A,B). Both markers show a trend toward a decreased number at 2 months of age (−15.8% for GFAP; −23% for Sox2) and a significant decrease at 8 months of age (−31% for GFAP, *p* = 0.0006; −30% for Sox2, *p* = 0.0061), suggesting that activation of the Shh pathway causes a progressive depletion of astrocytes in the ML of *Ptch1^+/−^* mice. Instead, we detected no differences in the number of cells expressing Iba1 in ML and DG between *Ptch1^+/−^* and WT mice (Supplementary Figure S1) suggesting that microglia is not influenced by overactivation of Shh signaling.

### Changes in the Expression of Neurogenesis-Related Genes in the DG of *Ptch1^+/−^* Mice

**Figure 4:**
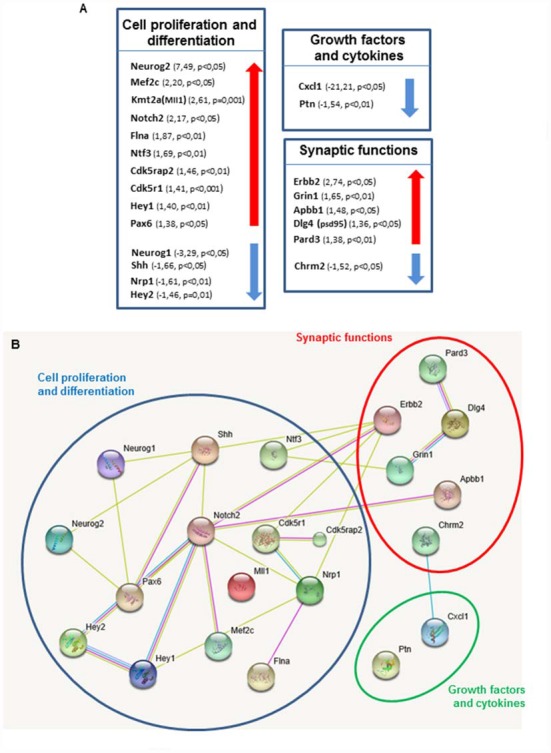
Shh pathway deregulation alters genes expression in key pathways in neurogenesis. **(A)** Each box contains genes showing altered expression in the DG of *Ptch1*^+/−^ male mice at 8 months of age. Arrows indicate direction of expression. A cut-off of 1.3 was applied for gene expression fold change. Altered genes are grouped in three categories: cell proliferation and differentiation, synaptic functions and growth factors and cytokines. **(B)** Predicted association between proteins based on observed patterns of simultaneous expression of genes. All deregulated genes were separately imported into STRING software.

Transcriptional control fulfills a key function in coordinating the developmental sequence of events that generate new functional neurons from stem cells. Given that Shh-dependent deregulation of neurogenesis in the DG is progressive we thought to investigate the underlying molecular mechanisms by analyzing possible differences between *Ptch1^+/−^* and WT mice in the expression profiles of 84 genes with established roles in neurogenesis at 8 months, when the defects are more severe. We carried out this analysis using a pathway-based PCR expression array in DG microdissected from 8 months old mice, i.e at the peak of Shh-dependent deregulation in the expression of stage-specific genetic program. In *Ptch1^+/−^* mice, 20% of the genes (17/84) were significantly upregulated and about 13.1% (11/84) downregulated (*p* ≤ 0.05; Supplementary Table S2). We considered a gene to have a significantly altered expression only if displaying a deregulation of 30% with a *p* value ≤ 0.05. In *Ptch1^+/−^* mice 15 genes (17.9%) fulfilled the above criteria for upregulation (i.e., *Apbb1, Cdk5r1, Cdk5rap2, Dlg4, Erbb2, Flna, Grin1, Hey1, Mef2c, Kmt2a, Notch2, Neurog2, Ntf3, Pard3* and *Pax6*), and 7 genes (8.3%) for downregulation (*Chrm2, Cxcl1, Hey2, Neurog1, Nrp1, Ptn and Shh*; Supplementary Table S2 in red and blue respectively, and Figure 4A). The top up- and down-regulated genes in *Ptch1^+/−^* mice were *Neurog2* (+7.2%) and *Cxcl1* (−21.21), respectively. We grouped deregulated genes in three categories: (a) cell proliferation/differentiation; (b) synaptic functions; and (c) growth factors and cytokines, as summarized in Figure 4A. We also show the predicted functional interaction network for the proteins encoded by the set of *Ptch1-*deregulated genes analyzed by STRING software (Figure 4B), demonstrating a high interconnectivity and highlighting many interacting components of the Notch signaling pathway.

On the whole, our findings show that the molecular network of Shh-dependent deregulated genes of the functional category of cell proliferation/differentiation converge on *Notch* pathway components (*Notch2; Hey1, Hey2; Neurog1; Neurog2* and *Pax6*), thus highlighting an important crosstalk between Notch and Shh signaling pathways.

### Shh Pathway Activation Induces DG Cells Proliferation by Increasing TLX Expression

**Figure 5:**
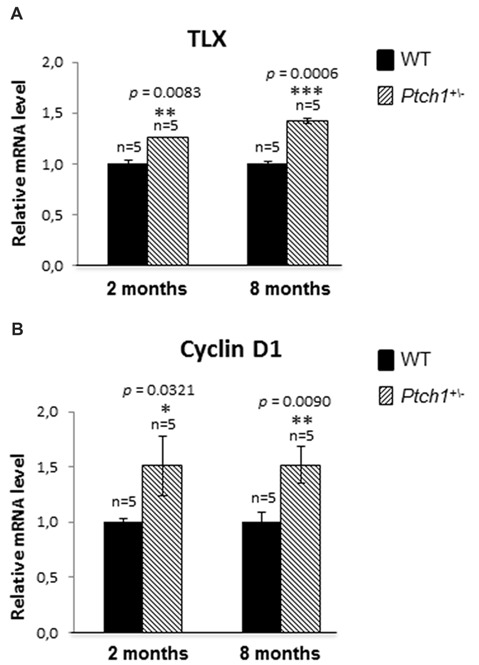
Shh pathway activation induces DG cells proliferation by increasing TLX and Cyclin D1 expression. **(A)** TLX mRNA relative expression in *Ptch1*^+/−^ mice at 2 and 8 months of age. **(B)** Cyclin D1 relative expression in *Ptch1*^+/−^ mice at 2 and 8 months. The number of mice used per test is indicated in the graphs (n). Data are reported as mean ± SEM *p < 0.05; **p < 0.01; ***p < 0.001 for comparison with controls (Student-*t* test).

Orphan nuclear receptor (TLX) is a central regulator of hippocampal neurogenesis balancing the maintenance of NSC populations with terminal neuronal differentiation (Shi et al., 2004; Elmi et al., 2010). As transgenic *TLX* expression in mice led to enlarged brains and elongated hippocampal DG (Murai et al., 2014), a phenotype strongly resembling those of the *Ptch1^+/−^* mice, we wondered whether expression of *TLX* might be modulated by Shh. Our qPCR analysis showed increased expression level of *TLX* mRNA, in *Ptch1^+/−^* mice both at 2 (1.3-fold, *p* = 0.0083) and 8 (1.4-fold, *p* = 0.0006) months of age, suggesting *TLX* expression dependence on the Shh signaling pathway (Figure 5A). As stimulation of NSCs proliferation by TLX is mediated by Cyclin D1 (Qu et al., 2010), we evaluated its expression in the DG by qPCR detecting a 1.5-fold increase in *Ptch1^+/−^* mice at both 2 (*p* = 0.0321) and 8 months (*p* = 0.0090) of age (Figure 5B).

Taken together our data suggest that *TLX* overexpression might concur to the defects in the morphology of the DG and in adult DG neurogenesis in *Ptch1^+/−^* mice through increased proliferation (Ki-67 and Cyclin D1, Figures 2G, 5B).

### Altered Behavior in *Ptch1^+/−^* Mice

**Figure 6:**
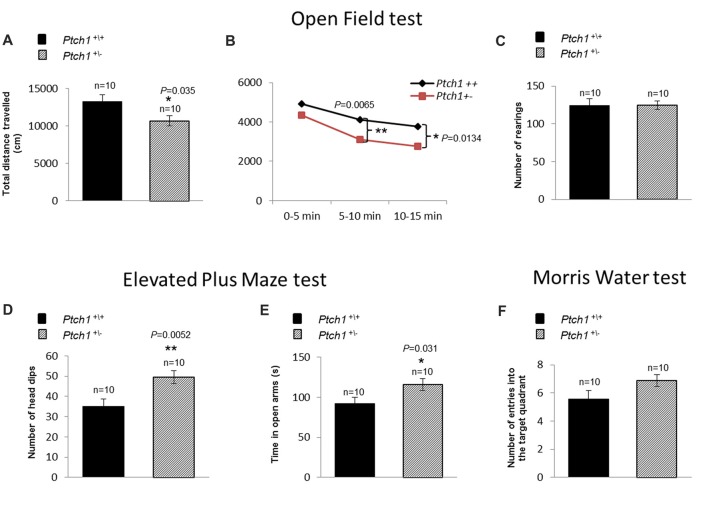
Shh pathway deregulation induces behavioral changes in *Ptch1^+/−^* mice. WT (*n* = 10) and *Ptch1*^+/−^ (*n* = 10) mice of 4 months of age were submitted to behavioral tests. **(A–C)** Open Field (activity and habituation). **(D,E)** Elevated Plus Maze (EPM; anxiety-like levels). **(F)** Water Maze (WM; spatial learning and memory). Data are given as the mean ± SEM. *p < 0.05; **p < 0.01. Statistical significance for all tests (described in the “*Cognitive Statistical Analysis*” paragraph) was established at *p* < 0.05.

There is growing appreciation of the prominent role of adult neurogenesis in hippocampus-dependent functions, and awareness that disturbance of adult hippocampal neurogenesis contributes to the pathophysiology of cognitive impairment. Given the Shh-dependent alterations in DG neurogenesis, we investigated the behavior of *Ptch1*^+/−^ mice at 4 months of age, through several tests such as OF (activity and habituation), EPM (anxiety-like levels), MWM (spatial learning and memory) and RAM test (spatial-working memory). Results from the OF test indicated that, compared to WT mice, *Ptch1*^+/−^ mice show a significant decrease in the total distance traveled, an indicator of activity levels (*p* = 0.035; Figure 6A). In addition, evaluation of habituation (total distance traveled in 15 min) show that *Ptch1*^+/–^ mice habituate earlier than WT animals (*p* = 0.0065; *p* = 0.0134), exhibiting an overall reduced activity (Figure 6B), although vertical activities (rearing) remain unchanged (Figure 6C). *Ptch1*^+/−^ mice also show a lower degree of anxiety-like behavior as illustrated by the results of the EPM test, showing increased number of head dips (*p* = 0.0052; Figure 6D) and the time spent in the open arms compared to WT mice (*p* = 0.031; Figure 6E). Instead, no effects on learning and memory performances has been assessed with the MWM test by measuring the number of crossing over the platform position in the target quadrant (Figure 6F) or with the RAM test (Supplementary Figure S2).

## Discussion

In this study, we investigated the *in vivo* consequences of Shh pathway constitutive activation on morphogenesis and neurogenesis of the DG, one of the two main neurogenic niches in the adult mammalian brain, and its impact on behavior. As *Ptch1* is the main Shh receptor, and considered a pathway antagonist, we used mice with *Ptch1* germline heterozygous deletion to genetically activate the pathway. Although Shh pathway is an important body size determinant, and *Ptch1^+/−^* mice often display overgrowth in various organs including CNS regions, alterations of the *Ptch1^+/−^* hippocampus have never been reported yet. To the best of our knowledge, we show here for the first time that Shh signaling activation in *Ptch1^+/−^* mice causes patterning defects in the DG, detectable as elongation and thinning of the blades. This is in fully agreement with the result of another study showing that the expression of a constitutively active Smo (SmoM2-YFP) resulted in a marked expansion of the DG, supporting a critical role for Shh signaling in the expansion and establishment of postnatal hippocampal progenitors (Han et al., 2008).

As many developmental pathways controlling morphogenesis are also critical for tissue homeostasis (Hodge et al., 2012), we investigated whether Shh signaling continues to act in the adult brain, when morphogenetic processes are concluded. Our results revealed that Shh pathway activation perturbs neurogenesis by altering the cell stage composition of the DG in *Ptch1^+/−^* mice. In agreement with the well-characterized mitogenic role of Shh, the first alterations detected in *Ptch1^+/−^* mice at 2 months of age were an increase in proliferation in the SGZ and a decreased density of mature neurons. With progressing age, alterations also included depletion of the quiescent NSCs, newborn/mature neurons and accumulation of TAPs, indicating that Shh activation induces progressive defects in the dynamic transition among the neural stages, by altering the balance between proliferative and neurogenic divisions. In agreement evidences from the literature show that activation of Shh signaling in *Ptch1^+/−^* mice critically alters the cell division mode in neural progenitors (Daynac et al., 2016) by enhancing symmetrical division of NSCs during cerebellar neurogenesis and in adult SVZ (Ferent et al., 2014; Yang et al., 2015). Instead, abrogation of Shh signaling through Smo removal compromises both proliferation and neurogenesis in the adult SVZ, permanently impairing the adult stem cell niche (Balordi and Fishell, 2007). Interestingly, our expression data identified Cdk5rap2 (centrosome-associated protein215) as a putative candidate involved in regulating changes in cell division mode. Mutations in this gene are responsible for the autosomal recessive microcephaly type3 and *Cdk5rap2* knockout mice display a changed orientation of the cleavage plane, leading to a switch from symmetric to asymmetric cell division during neurogenesis (Lizarraga et al., 2010). Although we detected increased *Cdk5rap2* expression during adulthood, a similar enhancement during DG morphogenesis might result in elongation and thinning of the DG in *Ptch1^+/−^* mice.

At the molecular level, DG from *Ptch1^+/−^* mice exhibited deregulation of several downstream components of Notch signaling pathway that plays a pivotal role in NSC maintenance and in the regulation of glial vs. neuronal identity. Many evidences demonstrate that Shh signaling can be modulated by the Notch pathway (Ringuette et al., 2014; Kong et al., 2015; Stasiulewicz et al., 2015). Instead, although not confirmed at the protein level, we here detected perturbations of key components of the Notch signaling pathway (*Notch2; Hey1/Hey2; Neurog1/Neurog2* and *Pax6*) in *Ptch1^+/−^* mice, demonstrating that Shh signaling may in turn influence Notch pathway activity and, therefore, suggesting a more complex interplay between these pathways. Similarly, conditional inactivation of *Ptch1* in the developing neocortex determined upregulation of Notch downstream targets that caused improper corticogenesis by increasing the number of symmetric proliferative divisions in Shh-activated VZ progenitors, that was reversed after attenuation of Notch signaling (Dave et al., 2011).

Unexpectedly, we found that the transcription factor Hes1, a canonical effector of Notch pathway and a molecular convergence point between Shh and Notch signaling in a number of different contexts (Ingram et al., 2008; Wall et al., 2009), was not deregulated in the hippocampus of *Ptch1^+/−^* mice. Instead, we detected deregulation of *Hey1* and *Hey2*, other essential Notch transducers, more prevalent in the cardiovascular system, but also involved in immune and neural functions, that may represent molecular hub between Shh and Notch signaling in the hippocampus. *Hey* genes encode inhibitory proteins, repressors of proneural proteins such as Neurogenins, controlling neuronal and glial fate determination, as well as cell type specification in many neural tissues. We also show that *Hey1*/*Hey2* deregulation was paralleled by a strong deregulation of *Neurog1* and *Neurog2*. Of note, *Neurog2*, the top upregulated gene in the DG of *Ptch1*^+/−^ mice, is positively regulated by Pax6, itself overexpressed (Scardigli et al., 2003), suggesting that a Shh-dependent deregulation of the expression of downstream components of the Notch pathway, such as Hey1/2 and Pax6, may promote self-renewal and repress differentiation of neuronal progenitors in the DG. Notably, *TLX* has been shown to be a direct target of Notch1/RBPJ in adult NSCs (Li et al., 2012) and to be positively regulated by Sox2 (Shimozaki et al., 2012; Islam et al., 2015). The primary TLX function in the developing and adult brain is to prevent the precocious differentiation of NSCs mainly through promotion of proliferation and inhibition of neurons differentiation (Shi et al., 2004; Li et al., 2008). *TLX* overexpression in TLX Tg mice leads to enhanced proliferation of neural progenitor cells and enlarged DG (Murai et al., 2014), features very similar to those observed in *Ptch1*^+/–^ mice. In this respect, the identification of *TLX* upregulation reported in this study suggests that the interplay between Shh and Notch signaling pathways may converge on TLX to regulate the balance between proliferative and neurogenic division during hippocampal neurogenesis.

Notwithstanding the upregulation of *Gli1* in the DG of *Ptch1*^+/−^ mice, indicative of Shh signaling activation, we show a downregulation of both Shh ligand and Nrp1, a positive regulator of the Shh pathway (Hillman et al., 2011), suggestive of an attempt to restrain Shh excess signaling. Notably, antagonistic interactions between Pax6 and Shh have been reported during spinal cord, telencephalon and diencephalic patterning (Fuccillo et al., 2006; Lek et al., 2010; Caballero et al., 2014), suggesting that *Pax6* overexpression may be central to Shh inhibition. *Pax6* upregulation also has implications for astrocytes differentiation, as forced *Pax6* expression in the cortex redirects astroglia towards neurogenesis (Heins et al., 2002; Blum et al., 2011), and indeed we detected a progressive depletion of astrocytes in the hippocampus of *Ptch1^+/−^* mice. Also, TLX represses astrocyte differentiation (Shi et al., 2004), suggesting that Shh-dependent upregulation of *Pax6* and *TLX* in the hippocampus might cause a shift from gliogenesis to neurogenesis. This may in turn, cause downregulation of the inflammatory chemokine Cxcl1, as cytokines/chemokines are mainly released by astrocytes. Consistent with our observations, Shh pathway inactivation through Smo ablation, or increase of Gli3 repressor form, cause reactive gliosis (Garcia et al., 2010; Petrova et al., 2013). All together these findings fully support a dependence on Shh signaling to prevent excessive astrocytes activation/differentiation and inflammatory processes in the adult brain.

In agreement with an emerging role of Shh signaling in differentiated neurons (Mitchell et al., 2012) and with the presence of Shh, Ptch1 and Smo at synapses (Petralia et al., 2011), suggestive of potential Shh roles in synaptic plasticity, we have also identified a number of deregulated synaptic function-related genes in the DG of *Ptch1^+/−^* mice (*Grin1, Dlg4, Apbb1, Chrm2*). However, the possibility exists that this deregulation may be an indirect consequence of altered neurogenesis, rather than a direct effect of Shh signaling. Similar deregulations are also found in human neurological pathologies such as Alzheimer and Parkinson’s Desease and the major depressive disorders (Hu et al., 2000; Leuba et al., 2014). Furthermore, decreased CHRM2 expression in cortex is associated with bipolar and major depressive disorders (Gibbons et al., 2009), and polymorphisms association studies support *CHRM2* involvement in major depressive disorder (Comings et al., 2002), potentially implying Shh pathway activation in synaptopathies and neurological pathologies in humans.

Notably, Shh-dependent morphological, cellular and molecular DG modifications, including depletion of newborn and mature neurons and synaptic function-related deregulation, were associated with altered behavior, as the *Ptch1^+/−^* mice exhibited hypoactivity and decreased anxiety-related behaviors in the OF and EPM tests but not alterations in MWM test. Importantly, TLX is a known regulator of cognitive and anxiety-related behaviors and hyperactivity has been documented in *TLX* knockout mice, pointing to a possible molecular mechanism involving *TLX* upregulation in behavioral deficit in *Ptch1^+/−^* mice (Zhang et al., 2008; O’Leary et al., 2016). Although anxiety-related behaviors are hippocampal-related function, contribution of *Ptch1*-dependent alterations in others brain areas to this phenotype cannot be excluded.

**Figure 7:**
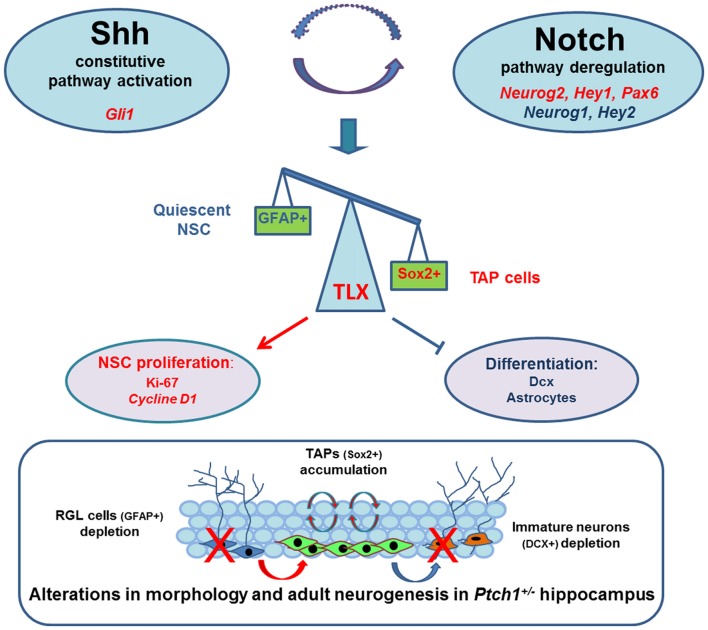
Schematic model in which Shh pathway activation positively feeds into the activities of the Notch signaling pathway resulting in upregulation of TLX, a direct target of Notch1/RBPJ, positively regulated by Sox2 and overexpressed in the DG of *Ptch1^+/−^* mice. TLX is a critical transcription factors for balancing neural stem cells (NSCs) maintenance with neuronal differentiation and its upregulation is likely to promote neurogenic division and repress terminal differentiation, leading to transit amplifyingprogenitor (TAP) cells accumulation and depletion of newborn neurons and astrocytes. The outcomes of this dysregulation are morphological and cellular and molecular defects in the hippocampus of *Ptch1^+/−^* mice that are fully compatible with the phenotype of TLX Tg mice showing DG elongation and enhanced proliferation of neural progenitors. Significantly up- and down-regulated genes are indicated in red and blue, respectively. Activation (*arrow lines)*; inhibition *(cut lines)*.

In summary to explain the Shh-dependent morphological and functional defects in DG of *Ptch1^+/−^* mice we propose a working model, depicted in Figure 7, in which Shh pathway activation positively feeds into the activities of the Notch signaling pathway, resulting in upregulation of TLX transcription factor, known to be critical for balancing NSCs proliferation and differentiation. Dysregulation of TLX might also concur to cause changes in inflammatory network and mouse behavior. Mechanistic understanding of cellular/molecular neurogenic process in health and disease is crucial for developing new treatments for a range of neurological conditions.

## Author Contributions

FA designed and conducted the major experiments, data analysis and drafted the manuscript. AC conducted part of the experiments and participated in data collection and drafting manuscript. MT and BT: collection and assembly of the data. AP: data analysis. ML-V, NS and MB: conducted behavioral tests and data analysis. AS: data interpretation and manuscript writing. SP: conception, design, data analysis, interpretation and manuscript writing.

## Conflict of Interest Statement

The authors declare that the research was conducted in the absence of any commercial or financial relationships that could be construed as a potential conflict of interest.
