# Supplementary material for: Alterations in Morphology and Adult Neurogenesis in the Dentate Gyrus of Patched1 Heterozygous Mice
Source: Front Mol Neurosci. 2018 May 23;11:168. doi: 10.3389/fnmol.2018.00168 (PMC5974030; doi:10.3389/fnmol.2018.00168)
Supplement: Supplementary file 1 [file Presentation_1.pdf]

Supplementary Material

Alterations in Morphology and Adult Neurogenesisin the Dentate Gyrus of *Patched1* Heterozygous Mice

Antonelli F., Casciati A., Tanori M., Tanno B., Linares-Vidal M.V., Serra N., Bellés M., Pannicelli A., Saran A., Pazzaglia S.*

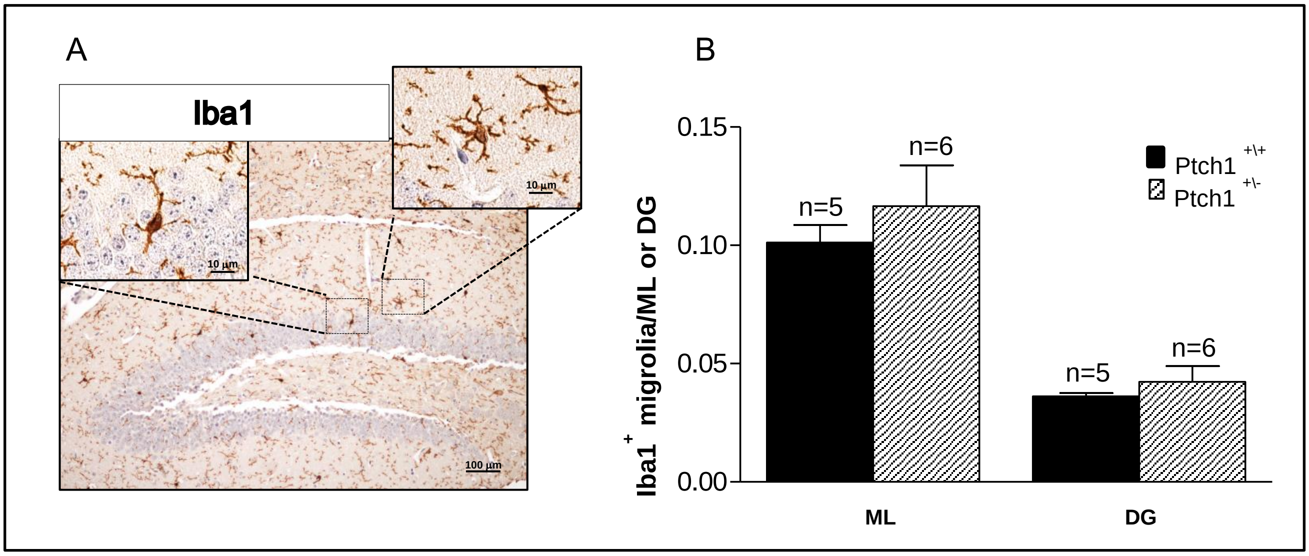

FIGURE S-1: Shh pathway deregulation does not affect microglia. (A) Immunostaining and (B) quantification of Iba1 positive microglia cells in the ML and DG of the hippocampus of 8-month old mice. Images, 10x magnification, scale bar = 100 µm; 100x magnification, scale bar = 10 µm. The number of mice used per test is indicated in the graph (n). Statistical analysis was determined using a two-tailed Student's t-test for comparison between pairs of means.

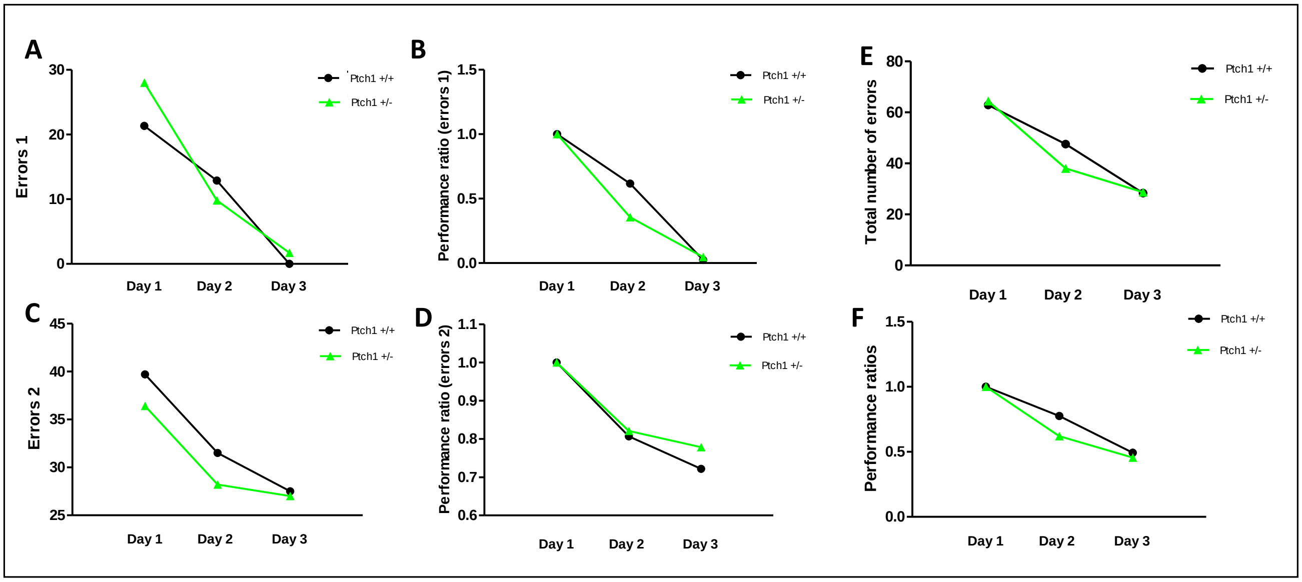

FIGURE S-2: Lack of effect of Shh signaling on spatial working memory using Radial Arm Maze (RAM) test at 4 months of age. *Ptch1*$^{+/+}$ (n=10) and *Ptch1*$^{+/-}$ (n=10) mice submitted to RAM test. (A) Errors type 1 (animal visits an arm and did not eat the reward). (B) Performance ratio 1 (errors type 1 standardized to day 1). (C) Errors type 2 (animal visits the same arm more than once during a single test session). (D) Performance ratio 2 (errors type 2 standardized to day 1). (E) Total number or errors (Errors 1 + Errors 2). (F) Performance ratios (total number of errors standardized to day 1). Data are given as the mean ± standard error (SEM). Statistical significance for all tests was established at $p < 0.05$.

TABLE S-1: Primer Sequences used for Real-Time Quantitative PCR.

Gene	Forward primer	Reverse primer
Gli1	5' – GAGGACCTGGAGAGAGAGGAGAA - 3'	5' – CCAGCGGCAGTCTGTCTCA – 3'
TLX	5' – CGATTAGACGCCACTGAA – 3'	5' – GGTATCTGGTATGAATGTAGC – 3'
Cyclin D1	5' – GCAAGCATGCACAGACCTT– 3'	5' – GTTGTGCGGTAGCAGGAGA – 3'

TABLE S-2: Expression changes in neurogenesis-related genes in *Ptch1*$^{+/-}$ compared to WT mice at 8 months of age. Significantly up- and down-regulated genes are indicated in red and blue, respectively. A cut-off of 1.3-fold in change of gene expression was applied.

Gene Symbol	Gene Title	Fold Regulation	p-value
Ache	Acetylcholinesterase	1,0193	0,801363
Adora1	Adenosine A1 receptor	-1,011	0,789168
Adora2a	Adenosine A2a receptor	1,7751	0,363635
Alk	Anaplastic lymphoma kinase	1,0263	0,84238
Apbb1	Amyloid beta (A4) precursor protein-binding, family B, member 1	1,4778	0,034366
Apoe	Apolipoprotein E	-1,041	0,699042
App	Amyloid beta (A4) precursor protein	2,1096	0,065162
Artn	Artemin	1,0093	0,949386
Ascl1	Achaete-scute complex homolog 1 (Drosophila)	-1,0559	0,659811
Bcl2	B-cell leukemia/lymphoma 2	-1,3134	0,114788
Bdnf	Brain derived neurotrophic factor	1,0287	0,649982
Bmp2	Bone morphogenetic protein 2	1,3263	0,966947
Bmp4	Bone morphogenetic protein 4	1,0838	0,396875
Bmp8b	Bone morphogenetic protein 8b	1,8556	0,054777
Cdk5r1	Cyclin-dependent kinase 5, regulatory subunit 1 (p35)	1,4105	0,000718
Cdk5rap2	CDK5 regulatory subunit associated protein 2	1,4586	0,007103
Chrm2	Cholinergic receptor, muscarinic 2, cardiac	-1,5223	0,026664
Creb1	CAMP responsive element binding protein 1	-1,2056	0,089482
Cxcl1	Chemokine (C-X-C motif) ligand 1	-21,214	0,016508
Dcx	Doublecortin	1,3752	0,066337
Dlg4	Discs, large homolog 4 (Drosophila)	1,3581	0,043013
Dll1	Delta-like 1 (Drosophila)	1,2717	0,351702
Drd2	Dopamine receptor D2	1,3107	0,590797
Dvl3	Dishevelled 3, dsh homolog (Drosophila)	1,0116	0,930631
Efnb1	Ephrin B1	1,2053	0,196411
Egf	Epidermal growth factor	-1,0915	0,386507
Ep300	E1A binding protein p300	1,4764	0,123412
Erbb2	V-erb-b2 erythroblastic leukemia viral oncogene homolog 2, neuro/glioblastoma derived oncogene homolog (avian)	2,7432	0,032266
Fgf2	Fibroblast growth factor 2	1,0482	0,639898
Flna	Filamin, alpha	1,8684	0,00437
Gdnf	Glial cell line derived neurotrophic factor	1,792	0,199044
Gpi1	Glucose phosphate isomerase 1	1,1516	0,044462
Grin1	Glutamate receptor, ionotropic, NMDA1 (zeta 1)	1,652	0,003729
Hdac4	Histone deacetylase 4	-1,413	0,091123
Hes1	Hairy and enhancer of split 1 (Drosophila)	-1,1447	0,072381
Hey1	Hairy/enhancer-of-split related with YRPW motif 1	1,3956	0,003629
Hey2	Hairy/enhancer-of-split related with YRPW motif 2	-1,4647	0,010087
Heyl	Hairy/enhancer-of-split related with YRPW motif-like	1,2233	0,372172
Il3	Interleukin 3	-1,2151	0,518317
Mdk	Midkine	1,0989	0,281474
Mef2c	Myocyte enhancer factor 2C	2,2004	0,023552
Kmt2a	Myeloid/lymphoid or mixed-lineage leukemia 1	2,6058	0,001
Map2	Microtubule-associated protein 2	1,0846	0,377087
Ndn	Necdin	-1,0582	0,649536
Ndp	Norrie disease (pseudoglioma) (human)	-1,2363	0,317091
Neurod1	Neurogenic differentiation 1	-1,1791	0,062632
Neurog1	Neurogenin 1	-3,2924	0,044035
Neurog2	Neurogenin 2	7,4938	0,0217
Nf1	Neurofibromatosis 1	1,4285	0,057908

Gene Symbol	Gene Title	Fold Regulation	p-value
Nog	*Noggin*	1,1322	0,340875
Notch1	*Notch gene homolog 1 (Drosophila)*	1,7943	0,076791
Notch2	*Notch gene homolog 2 (Drosophila)*	2,1685	0,012814
Nr2e3	*Nuclear receptor subfamily 2, group E, member 3*	1,4149	0,134383
Nrcam	*Neuron-glia-CAM-related cell adhesion molecule*	-1,3396	0,051273
Nrg1	*Neuregulin 1*	1,0164	0,796512
Nrp1	*Neuropilin 1*	-1,6076	0,006221
Nrp2	*Neuropilin 2*	-1,0567	0,587594
Ntf3	*Neurotrophin 3*	1,6855	0,004428
Ntn1	*Netrin 1*	1,5268	0,099107
Tenm1	*Odd Oz/ten-m homolog 1 (Drosophila)*	-1,1639	0,476195
Olig2	*Oligodendrocyte transcription factor 2*	-1,5311	0,08057
Pafah1b1	*Platelet-activating factor acetylhydrolase, isoform 1b, subunit 1*	1,0365	0,644714
Pard3	*Par-3 (partitioning defective 3) homolog (C. elegans)*	1,372	0,006458
Pax3	*Paired box gene 3*	-1,2866	0,490475
Pax5	*Paired box gene 5*	2,2816	0,08455
Pax6	*Paired box gene 6*	1,3846	0,039007
Pou3f3	*POU domain, class 3, transcription factor 3*	1,4539	0,156044
Pou4f1	*POU domain, class 4, transcription factor 1*	1,2213	0,416185
Ptn	*Pleiotrophin*	-1,5426	0,002388
Rac1	*RAS-related C3 botulinum substrate 1*	-1,147	0,022048
Robo1	*Roundabout homolog 1 (Drosophila)*	-1,2136	0,030484
Rtn4	*Reticulon 4*	-1,2882	0,043584
S100a6	*S100 calcium binding protein A6 (calcyclin)*	-1,0518	0,537386
S100b	*S100 protein, beta polypeptide, neural*	1,0582	0,075549
Shh	*Sonic hedgehog*	-1,6562	0,038125
Slit2	*Slit homolog 2 (Drosophila)*	1,062	0,809289
Sod1	*Superoxide dismutase 1, soluble*	-1,206	0,002587
Sox2	*SRY-box containing gene 2*	-1,0306	0,248389
Sox3	*SRY-box containing gene 3*	1,629	0,15878
Stat3	*Signal transducer and activator of transcription 3*	1,1534	0,126858
Tgfb1	*Transforming growth factor, beta 1*	-1,0847	0,454668
Th	*Tyrosine hydroxylase*	ND	ND
Tnr	*Tenascin R*	1,1983	0,039991
Vegfa	*Vascular endothelial growth factor A*	-1,0553	0,777697
